# The relationships among endurance performance measures as estimated from VO_2PEAK_, ventilatory threshold, and electromyographic fatigue threshold: a relationship design

**DOI:** 10.1186/1476-5918-7-15

**Published:** 2008-09-10

**Authors:** Jennifer L Graef, Abbie E Smith, Kristina L Kendall, Ashley A Walter, Jordan R Moon, Christopher M Lockwood, Travis W Beck, Joel T Cramer, Jeffrey R Stout

**Affiliations:** 1 Department of Health and Exercise Science, University of Oklahoma, Huston Huffman Center, 1401 Asp Ave., Norman, OK 73019, USA

## Abstract

**Background:**

The use of surface electromyography has been accepted as a valid, non-invasive measure of neuromuscular fatigue. In particular, the electromyographic fatigue threshold test (EMG_FT_) is a reliable submaximal tool to identify the onset of fatigue. This study examined the metabolic relationship between VO_2PEAK_, ventilatory threshold (VT), and the EMG_FT_, as well as compared the power output at VO_2PEAK_, VT, and EMG_FT_.

**Methods:**

Thirty-eight college-aged males (mean ± SD = 22.5 ± 3.5 yrs) performed an incremental test to exhaustion on an electronically-braked cycle ergometer for the determination of VO_2PEAK _and VT. Each subject also performed a discontinuous incremental cycle ergometer test to determine their EMG_FT _value, determined from bipolar surface electrodes placed on the longitudinal axis of the vastus lateralis of the right thigh. Subjects completed a total of four, 2-minute work bouts (ranging from 75–325 W). Adequate rest was given between bouts to allow for subjects' heart rate to drop within 10 beats of their resting heart rate. The EMG amplitude was averaged over 10-second intervals and plotted over the 2-minute work bout. The resulting slopes from each successive work bout were used to calculate EMG_FT_.

**Results:**

Power outputs and VO_2 _values from each subject's incremental test to exhaustion were regressed. The linear equations were used to compute the VO_2 _value that corresponded to each fatigue threshold. Two separate one-way repeated measure ANOVAs indicated significant differences (p < 0.05) among metabolic parameters and power outputs. However, the mean metabolic values for VT (1.90 ± 0.50 l·min^-1^) and EMG_FT_VO_2_(1.84 ± 0.53 l·min^-1^) were not significantly different (p > 0.05) and were highly correlated (r = 0.750). Furthermore, the mean workload at VT was 130.7 ± 37.8 W compared with 134.1 ± 43.5 W at EMG_FT _(p > 0.05) with a strong correlation between the two variables (r = 0.766).

**Conclusion:**

Metabolic measurements, as well as the power outputs at VT and EMG_FT_, were strongly correlated. The significant relationship between VT and EMG_FT _suggests that both procedures may reflect similar physiological factors associated with the onset of fatigue. As a result of these findings, the EMG_FT _test may provide an attractive alternative to estimating VT.

## Background

Matsumoto et al. [1] and Moritani et al. [2] have proposed an incremental cycle ergometer test utilizing fatigue curves to identify the maximal power output at which an individual can maintain without evidence of fatigue, described as the electromyographic fatigue threshold (EMG_FT_). The EMG_FT _test is an adaptation to deVries' [3] original monopolar physical working capacity at the fatigue threshold (PWC_FT_) test, using a bipolar supramaximal protocol. The EMG_FT _involves determining the rate of rise in electrical activity from the vastus lateralis during four, two-minute work bouts on a cycle ergometer, with varying power outputs. It has been suggested that the rise in electrical activity is a result of progressive recruitment of additional motor units (MU) and/or an increase in the firing frequency of MUs that have already been recruited. Several investigations have used surface electromyography to characterize the fatigue-induced increase in EMG amplitude, as well as to identify the power output associated with the onset of neuromuscular fatigue during cycle ergometry [1,2,4-8]. Matsumoto et al. [1] described the EMG_FT _as the highest intensity sustainable on a cycle ergometer without signs of neuromuscular fatigue. In addition, Moritani et al. [2] suggested a strong physiological link between myoelectrical changes at fatigue and anaerobic threshold. Furthermore, the EMG_FT _method has been reported as a valid and reliable technique for examining the transition from aerobic to anaerobic metabolism during exercise [4,6,7]. Identifying a reliable, non-invasive way to measure and predict the onset of fatigue has potential use in clinical populations, as well as serving as a training tool for those with minimal testing equipment. Therefore, the purpose of this study was to examine the metabolic relationship between VO_2PEAK_, ventilatory threshold (VT), and the EMG_FT_, as well as to compare the power output at VO_2PEAK_, VT, and EMG_FT_.

## Methods

### Participants

Thirty-eight recreationally trained (1–5 hours/week), college-aged men (Table 1) volunteered to participate in this study. All procedures were approved by the University of Oklahoma Institutional Review Board for Human Subjects, and written informed consent was obtained from each participant prior to any testing.

**Table 1:** Descriptive statistics (mean ± SD) of the subjects.

	Subjects (n = 35)
Age (yrs)	22.6 ± 3.5
Height (cm)	177.1 ± 7.1
Weight (kg)	77.0 ± 11.0

### Determination of VO_2PEAK _and Ventilatory Threshold

Participants performed a continuous graded exercise test (GXT) on an electronically-braked cycle ergometer (Corival Lode 400, Groningen, The Netherlands) to determine maximal oxygen consumption (VO_2PEAK_) and ventilatory threshold (VT). Following a five-minute warm-up (50 W), the workload was increased 25 W every two minutes until the participants were unable to maintain 70 rpm, or until volitional fatigue.

Ventilatory threshold was determined as a plot of ventilation (V_E_) vs. oxygen consumption (VO_2_), as described previously [9]. Two linear regression lines were fit to the lower and upper portions of the V_E _vs. VO_2 _curve before and after the break points, respectively. The intersection of these two lines was defined as VT.

### Gas Exchange Analysis

Open circuit spirometry was used to analyze the gas exchange data using the Parvo-Medics TrueOne 2400^® ^Metabolic Measurement System (Sandy, Utah, United States). Oxygen and carbon dioxide were analyzed through a sampling line after the gases passed through a heated pneumotach and mixing chamber. The data were averaged over 15-second intervals. The highest average VO_2 _value during the GXT was recorded as the VO_2PEAK _if it coincided with at least two of the following criteria: (a) a plateau in heart rate (HR) or HR values within 10% of the age-predicted HRmax, (b) a plateau in VO_2 _(defined by an increase of no more than 150 ml·min^-1^), and/or (c) an RER value greater than 1.15 [10].

### Electromyography

Pre-gelled bipolar (2.54 cm center-to-center) surface electrodes (Ag-Ag Cl, Quinton Quick Prep, Quinton Instruments Co., Bothell, WA) were placed over the lateral portion of the vastus lateralis muscle, midway between the greater trochanter and the lateral condyle of the femur. A reference electrode was placed over the 7^th ^cervical vertebrae. The raw EMG signals were pre-amplified ((gain × 1,000) EMG 100C, Biopac Systems, Inc., Santa Barbara, CA), sampled at 1,000 Hz and bandpass filtered from 10–500 Hz (zero-lag 8^th ^order Butterworth filter). All EMG amplitude values were stored on a personal computer (Dell Inspiron 8200, Dell, Inc., Round Rock, TX) and analyzed off-line using custom-written software (LabVIEW v 7.1, National Instruments, Austin, TX).

### Determination of the EMG_FT_

**Figure 1:**
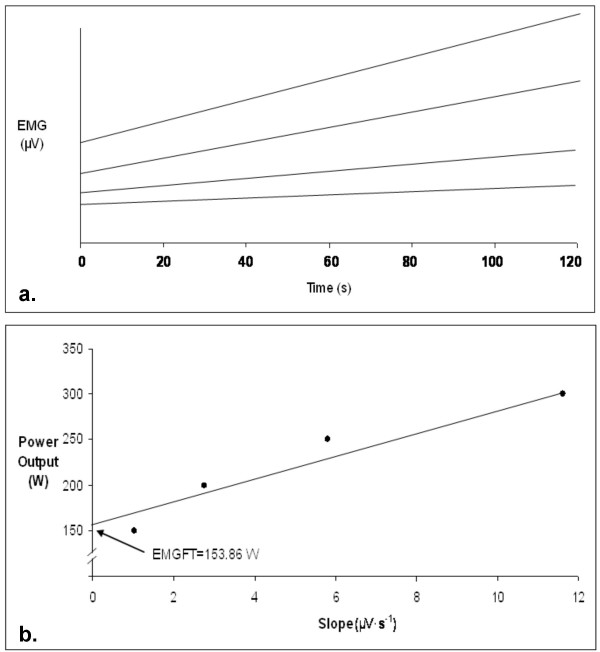
**Determination of EMG_FT_**. **a**. Describes the relationship between EMG amplitude and time for the four power outputs used in the EMG_FT _test. The greatest slope was a result from the highest power output. **b**. Depicts the relationship for the power outputs versus slope coefficients with the y-intercept defined as the EMG_FT_.

Participants returned 24–48 hours after the GXT to perform the EMG_FT _test. Following a five-minute warm-up on an electronically-braked cycle ergometer (Quinton Corival 400), participants completed four two-minute cycling bouts at incrementally ascending workloads (75 W–300 W). The initial workload corresponded with the workload at which VT occurred, determined during the GXT. Adequate rest was given between bouts to allow for participants' heart rate to drop within 10 beats of their resting heart rate. The rate of rise in EMG amplitude values (EMG slope) from the four workloads were plotted over 120 seconds (Figure 1a). The EMG slope values for each of the four power outputs were then plotted to determine EMG_FT _(Figure 1b). The line of best fit was extrapolated to the y-axis, and the power output at which it intersected the y-axis was defined as the EMG_FT_. The participants completed the EMG_FT _protocol two times; familiarization trial and baseline.

Test-rest reliability for the EMG_FT _protocol, determined at the University of Oklahoma, resulted in an intraclass correlation coefficient (ICC) of 0.935 (SEM 5.03 W). The ICC from this lab was higher than previously reported using the vastus lateralis (ICC = 0.65) [11].

### Statistical Analysis

Each participant's power outputs from the EMG_FT _and the VO_2PEAK _corresponding to the outputs during the GXT were regressed. A linear equation was developed to predict the VO_2 _value that corresponded to the EMG_FT _(EMG_FT_VO_2_). A one-way repeated measures ANOVA was used to determine differences between the EMG_FT_VO_2_, VT, and VO_2PEAK_. When appropriate, follow-up dependent t-test analyses were run. Correlation analyses were run to determine the strength of the relationship between EMG_FT _vs. VT (watts) and EMG_FT_VO_2 _vs. VT (l·min^-1^). All data are reported as mean ± S.E.

## Results

**Figure 2:**
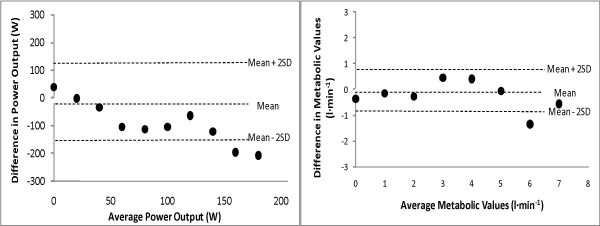
**Comparison of EMG_FT _and VT**. The relationship between differences in EMG_FT _and VT mean power outputs (W) and metabolic values (l·min^-1^).

A one-way repeated measures analysis of variance (ANOVA) indicated a significant (p < 0.001) difference among metabolic parameters for EMG_FT_VO_2_, VT, and VO_2PEAK_. Table 2 presents the mean metabolic and power output values for EMG_FT _and VT, as well as the correlation coefficients for these variables. Dependent t-test analyses resulted in no significant differences (p = 0.794) between the power output at which EMG_FT _and VT occurred, as well as no significant differences (p = 0.204) between the EMG_FT_VO_2 _and VT. However, the VO_2PEAK _values were significantly different from both parameters. Furthermore, power output and metabolic parameters for EMG_FT _and VT were strongly correlated (r = 0.766 and r = 0.750, respectively). Figure 2 displays the relationship between EMG_FT _and VT parameters for mean power output (W) and metabolic values (l·min^-1^). Based on significant correlation analysis (Table 2), a regression equation was developed to predict VT from EMG_FT _which resulted in a strong relationship with a low (less than 4% of mean) standard error of estimate (SEE):

**Table 2:** Mean ± standard error (SE) values and correlations for EMG_FT _and VT.

			**Correlation analysis**	
			
	**Mean ± SEM (l-min^-1^)**	**Mean ± SEM (W)**	**EMG_FT_(l-min^-1^)**	**EMG_FT_(W)**
Electromyographic Fatigue Threshold	1.84 ± 0.09	134.11 ± 7.06	1.000	1.000
Ventilatory Threshold	1.89 ± 0.08	130.71 ± 6.13	0.750*	0.766*

*p < 0.01

VT (W) = 0.665(EMG_FT_) + 41.53; SEE = 13 W

## Discussion

The results of the present study demonstrated support for previous work verifying the use of the EMG_FT _as a reliable and non-invasive method for identifying the onset of neuromuscular fatigue [1-7]. In addition, a highly significant relationship between power output values at EMG_FT _and VT was found. Furthermore, no significant difference between metabolic values at EMG_FT_VO_2 _and VT was found. Several studies have suggested the use of the EMG_FT _as a simple and attractive alternative to identify the onset of fatigue [1-3,6,7,12]. The results of the current study further support the myoelectrical and physiological similarities proposed between the EMG_FT _and VT.

The EMG_FT _theoretically represents the highest power output that can be sustained without electromyographic evidence of neuromuscular fatigue [1,2]. In addition, the VT has been proposed to correlate with a workload that theoretically can be maintained without evidence of fatigue [7]. The VT may be an indicator of the ability of the cardiovascular system to adequately supply oxygen to the working muscles to prevent muscle anaerobisis [13]. Performing exercise at an intensity greater than the VT would result in an inadequate supply of oxygen to the working muscle, resulting in the recruitment of Type II muscle fibers, quickly leading to fatigue [13]. The fatigued state of a muscle has been associated with changes in motor unit recruitment and/or changes in the frequency of motor unit firing resulting in an increase in EMG activity [8]. Several studies have proposed a strong physiological relationship between VT and the onset of neuromuscular fatigue, with both measures representing recruitment of Type II muscle fibers due to the transition from aerobic to anaerobic metabolism [3,4,6,8,14]. As a result, there would be an increase in muscle lactate concentration corresponding to a decrease skeletal muscle pH, which may further signal arterial chemoreceptors that alter ventilatory regulating mechanisms [15-17]. The evidence presented in this study suggests that the EMG_FT _and VT may reflect similar acute physiological adaptations that occur during exercise.

The data in the present study are in agreement with previous investigations that have reported VT and EMG_FT _to occur at similar power outputs during cycle ergometry [1,3,7,8,12]. In addition, the current study provides new data indicating no significant difference between the VT and EMG_FT_VO_2_. In contrast, Moritani et al. determined EMG_FT_VO_2 _by calculating each participant's delta mechanical efficiency values [2], as described by Gaesser and Brooks [18], during the incremental exercise test. Although Moritani et al. reported a significant difference between VT and EMG_FT_VO_2 _using the delta mechanical efficiency technique, Gaesser and Brooks determined that this technique was not valid. However, the significant relationships (Table 2) between VT vs. EMG_FT _and VT vs. EMG_FT_VO_2 _found in the present study suggest the possibility of using EMG_FT_, rather than gas analysis, to predict VT. Based on this assumption, a regression equation was developed to predict VT from EMG_FT_: VT (W) = 0.665(EMG_FT_) + 41.53; SEE = 13 W. The strong correlation and low prediction error (SEE < 4.0%) indicate that the EMG_FT _test may be an alternative and salient method to predict VT.

## Conclusion

In summary, the relationship between VT and EMG_FT_VO_2 _suggests a possible attractive alternative to measuring VT via gas analysis. Determining VT using gas analysis requires participants to reach volitional fatigue during a graded exercise test, and, therefore, the results may be influenced by motivation. The EMG_FT _test consists of submaximal workloads which should eliminate the influence of participant motivation. In addition, due to the submaximal nature of the test, it may provide a safe alternative to determining VT for clinical populations in which maximal exertion may not be safe. Furthermore, the EMG_FT _test may reduce or eliminate discomfort experienced during gas analysis due to the gas measurement equipment. However, additional studies are needed to validate the regression equation proposed in the present study to predict VT using EMG_FT_. In addition, future studies are warranted to determine whether the regression equation can accurately track changes in VT over time with training.

## Competing interests

The authors declare that they have no competing interests.

## Authors' contributions

JG, AS, and KK contributed in writing and editing the manuscript along with concept and design, data acquisition, and data analysis and interpretation. AW and CL contributed in concept and design, data acquisition, and data analysis and interpretation. JM, TB, JC, and JS contributed in writing and editing the manuscript, as well as concept and design. All authors have read and approved the final manuscript.

## References

[B1] Matsumoto T, Ito K, Moritani T (1991). The relationship between anaerobic threshold and electromyographic fatigue threshold in college women. Eur J Appl Physiol Occup Physiol.

[B2] Moritani T, Takaishi T, Matsumoto T (1993). Determination of maximal power output at neuromuscular fatigue threshold. J Appl Physiol.

[B3] deVries HA, Moritani T, Nagata A, Magnussen K (1982). The relation between critical power and neuromuscular fatigue as estimated from electromyographic data. Ergonomics.

[B4] Lucia A, Sanchez O, Carvajal A, Chicharro JL (1999). Analysis of the aerobic-anaerobic transition in elite cyclists during incremental exercise with the use of electromyography. Br J Sports Med.

[B5] Hanon C, Thepaut-Mathieu C, Hausswirth C, Le Chevalier JM (1998). Electromyogram as an indicator of neuromuscular fatigue during incremental exercise. Eur J Appl Physiol Occup Physiol.

[B6] Hug F, Faucher M, Kipson N, Jammes Y (2003). EMG signs of neuromuscular fatigue related to the ventilatory threshold during cycling exercise. Clin Physiol Funct Imaging.

[B7] Maestu J, Cicchella A, Purge P, Ruosi S, Jurimae J, Jurimae T (2006). Electromyographic and neuromuscular fatigue thresholds as concepts of fatigue. J Strength Cond Res.

[B8] Nagata A, Muro M, Moritani T, Yoshida T (1981). Anaerobic threshold determination by blood lactate and myoelectric signals. Jpn J Physiol.

[B9] Orr GW, Green HJ, Hughson RL, Bennett HG (1982). A computer linear regression model to determine ventilatory anaerobic threshold. J Appl Physiol.

[B10] Day JR, Rossiter HB, Coats EM, Skasick A, Whipp BJ (2003). The maximally attainable VO2 during exercise in humans: the peak vs. maximum issue. J Appl Physiol.

[B11] Pavlat DJ, Housh TJ, Johnson GO, Schmidt RJ, Eckerson JM (1993). An examination of the electromyographic fatigue threshold test. Eur J Appl Physiol Occup Physiol.

[B12] Helal JN, Guezennec CY, Goubel F (1987). The aerobic-anaerobic transition: re-examination of the threshold concept including an electromyographic approach. Eur J Appl Physiol Occup Physiol.

[B13] Wasserman K, Beaver WL, Whipp BJ (1990). Gas exchange theory and the lactic acidosis (anaerobic) threshold. Circulation.

[B14] Skinner JS, McLellan TH (1980). The transition from aerobic to anaerobic metabolism. Res Q Exerc Sport.

[B15] Jorfeldt L, Juhlin-Dannfelt A, Karlsson J (1978). Lactate release in relation to tissue lactate in human skeletal muscle during exercise. J Appl Physiol.

[B16] Sahlin K, Katz A, Henriksson J (1987). Redox state and lactate accumulation in human skeletal muscle during dynamic exercise. Biochem J.

[B17] Vogiatzis I, Spurway NC, Jennett S, Wilson J, Sinclair J (1996). Changes in ventilation related to changes in electromyograph activity during repetitive bouts of isometric exercise in simulated sailing. Eur J Appl Physiol Occup Physiol.

[B18] Gaesser GA, Brooks GA (1975). Muscular efficiency during steady-rate exercise: effects of speed and work rate. J Appl Physiol.

